# The impact of using primaquine without prior G6PD testing: a case series describing the obstacles to the medical management of haemolysis

**DOI:** 10.12688/wellcomeopenres.15100.2

**Published:** 2019-04-09

**Authors:** Cindy S. Chu, Germana Bancone, Nay Lin Soe, Verena I. Carrara, Gornpan Gornsawun, François Nosten

**Affiliations:** 1 Shoklo Malaria Research Unit, Mahidol-Oxford Tropical Medicine Research Unit, Faculty of Tropical Medicine, Mahidol University, Mae Sot, Thailand; 2 Centre for Tropical Medicine and Global Health, Nuffield Department of Medicine, University of Oxford, Oxford, UK; 3 Department of Medicine, Swiss Tropical and Public Health Institute, Basel, Switzerland

**Keywords:** Plasmodium vivax, Primaquine, Haemolysis, G6PD deficiency

## Abstract

Radical cure of *Plasmodium vivax* malaria in glucose-6-phosphate dehydrogenase (G6PD) deficient individuals employs weekly primaquine dosing. This is the only recommended regimen for this patient sub-group. If national malaria programs mandate daily primaquine dosing (the recommended regimen for G6PD normal individuals), then G6PD testing before prescription is necessary to avoid iatrogenic haemolysis in G6PD deficient individuals. In this case series, two *P. vivax* infected patients with unknown G6PD status from two different countries were prescribed primaquine as per national malaria program guidelines. During treatment both patients presented to the clinic with symptoms of anaemia after taking primaquine incorrectly. The clinical management of the iatrogenic severe haemolysis that occurred in these patients demonstrates the various adverse effects primaquine can cause, that other common medical treatments also have haemolytic potential, and how the diagnosis of G6PD deficiency can be elusive during acute haemolysis.

Health care providers should provide careful instructions about primaquine dosing, be watchful for haemolysis, and have a high index of suspicion for G6PD deficiency in the presence of haemolysis if the G6PD status is previously unknown.

## Introduction

Primaquine is the only treatment available for the radical cure of *Plasmodium vivax* malaria. It is not widely used because G6PD deficient persons develop a dose dependent haemolysis when exposed to the daily doses in the 14-day regimen. They should receive the weekly dose of 0.75 mg/kg in the 8-week regimen which delivers nearly the same total dose. The evidence supporting the safety of the 8-week regimen was derived from a study performed in the 1960’s where a weekly primaquine dose (45 mg) caused less haemolysis than a daily dose (30 mg) in G6PD deficient adult patients of African descent ^1^. The efficacy of this weekly dose was 90% and this resulted in the recommendation that 45 mg or 0.75mg/kg/week for 8 weeks be used in G6PD deficient persons within the general population “providing some supervision of drug administration be maintained” ^1^. Contemporary studies show that the efficacy of this dose remains over 90% ^2^ and more safety assessments are needed ^3^.

In some areas, the practice is to prescribe primaquine to patients in the entirety of the dose without G6PD testing and without further follow up or medical supervision. Adherence to this prolonged weekly treatment regimen is an issue especially if common adverse effects such as abdominal pain occur. In Myanmar, the national policy up to 2018 is to give the 8-week 0.75mg base/kg regimen to all *P. vivax* infected patients. In Thailand, the 14-day 0.25 mg base/kg/day regimen is given. Testing for G6PD deficiency prior to treatment is not a requirement in the national policy for malaria treatment in either country. This means that undiagnosed G6PD deficient patients have the potential to develop primaquine induced haemolysis, especially with daily regimens ^4^. The haemolysis can be more severe if doses are taken incorrectly with either primaquine regimen ^5^.

The prevalence of G6PD deficiency varies by geographic region and between ethnic groups ^6^. In Thailand, it reaches 20% in some populations ^7, 8^. In neighbouring Myanmar the prevalence ranges from 4% to 12% in the general population ^9^ and nearly 13% in males living in eastern Myanmar along the Thailand border ^10, 11^. A substantial proportion of populations are thus at risk for primaquine induced haemolysis if G6PD deficiency is not diagnosed before treatment ^12^ or if primaquine is taken incorrectly.

Here, we describe primaquine induced haemolysis in two G6PD Mahidol hemizygous males. The first case received the initial *P. vivax* diagnosis and treatment in Myanmar and the second case in Thailand.

## Case 1

A 16-year-old male presented to the clinic. He was weak and nearly prostrate. He had a >10 day history of fever, dizziness, cough, dyspnoea and abdominal pain. Earlier in the morning, he had passed red urine. He was diagnosed with *P. vivax* malaria at a government clinic in Myanmar 10 days previous. In addition to chloroquine, he was prescribed a course (number of tablets unknown) of primaquine tablets (7.5 mg) with verbal instructions from the health worker. This was presumed to be the 8-week primaquine regimen as per the Myanmar national malaria policy. At home, the patient took 30mg daily for 4 days (1mg/kg/day). He stopped because he felt unwell.

On arrival, the Glasgow Coma Score (GCS) score was 15/15. He appeared severely unwell and was unable to speak due to dyspnoea. Weight was 30 kg, temperature 37.5°C, heart rate (HR) 113 beats per minute, respiratory rate (RR) 36 breaths per minute, blood pressure (BP) 90/50 mmHg, and oxygen saturation (SaO2) 82–87% on 5LO2 by face mask. On physical examination the following were noted: icteric sclerae, conjunctival pallor, tachycardia with normal heart sounds and no audible murmur, clear lung sounds bilaterally, a soft abdomen with no hepatosplenomegaly, and pallor of the hands.

Initial blood work was performed ( Table 1); malaria smear was negative, haematocrit was 15%, and G6PD fluorescent spot test (FST) was normal (not deficient). He was resuscitated with normal saline and treated empirically with ceftriaxone 1gm intravenously. Within 4 hours of arrival, the patient was given one unit of blood to which he responded well. The donor was a female whose G6PD status was normal by FST and genotyping. Vital signs after blood transfusion were: HR 90 beats per minute, RR 24 breaths per minute, BP 100/50 mmHg, and SaO2 90% on 2LO2 by nasal cannula. Urine output was normal throughout the resuscitation period.

**Table 1.** Laboratory test results for Cases 1 and 2.

Laboratory test (reference range)	Case 1 (fever history >10 days)			Case 2 (fever history 4 days)				
	Day 1 *	Day 2	Day 8	Day 1	Day 2	Day 3 *	Day 4	Day 8
Field Haematocrit (36–56%) ^a^	15	20	30	34	30	27	24	24
Methaemoglobin (<3%)	-	-	-	17.1	18.3	15.5	2.7	0.6
RBC count (3.80–5.30 x10 ^6^/uL)	1.43	-	-	5.01	-	3.57	-	-
Haemoglobin (11.0–17.0 g/dL)	4.1	-	-	9.8	-	6.8	-	-
MCV (80–100 fL)	93.7	-	-	63.1	-	65.0	-	-
WBC (4.0–9.0 x10 ^3^/uL)	40.4	-	-	5.5	-	11.3	-	-
Neutrophil (1.7–7.7 x10 ^3^/uL)	30.8	-	-	4.3	-	7.3	-	-
Lymphocyte (0.4–4.4 x10 ^3^/uL)	5.4	-	-	0.9	-	2.3	-	-
Platelets (120–380 x10 ^3^/uL)	596	-	-	224	-	352	-	-
Nucleated RBC (<1%)	9	-	-	-	-	-	-	-
Reticulocyte count (<2.5%)	ND	-	-	0.5	-	1.2	-	-
G6PD FST (normal)	normal	-	-	deficient	-	-	-	-
G6PD spectrophotometry (>5.6 IU/gHb)	-	-	-	0.39	-	-	-	-
G6PD genotype ^b^	-	-	Mahidol	-	-	-	-	Mahidol
Urine colour (clear or yellow)	red	-	clear	yellow/red	-	black	-	clear
Urine stick for haemoglobin ^c^	-	4+	-	4+	4+	-	-	-
CRP (<8 mg/L)	89.6	-	-	<8	-	6.4	-	-
BUN (6–20 mg/dL)	35	-	-	-	12	11	17	-
Creatinine (0.67–1.17 mg/dL)	1.22	-	-	-	0.58	0.64	0.97	-
LDH (135–225 IU/L)	5,381	-	-	-	-	-	-	-
Dengue rapid diagnostic test ^c^	negative	-	-	-	-	negative	-	-
Scrub rapid diagnostic test ^c^	-	-	-	-	-	negative	-	-

* A blood transfusion was given on this day and the blood work results are prior.

^a^ Capillary sample is obtained, centrifuged, and read manually

^b^ G6PD genotyping was performed only for Mahidol variant

^c ^For these tests the normal result is ‘negative’

The patient’s clinical condition improved daily for the remainder of the hospital course. The DNA analysis showed that the patient was hemizygote for G6PD Mahidol variant. On the 8 ^th^ day of hospitalisation, the patient was discharged home. His haematocrit had increased to 30%. The patient was counselled not to take daily doses of primaquine, but that the 8-week course under supervision would be possible. He was given a ‘G6PD deficiency card’ describing the disease and drugs to avoid, which was to be presented at all future health care visits. It was emphasised that other members of the family might also have G6PD deficiency and should be tested. Weekly primaquine dosing for the radical cure of *P. vivax* malaria could not be prescribed because close medical supervision during treatment was not possible. Health care workers with specific knowledge of haemolysis and access to hospital care were not available in their remote village.

## Case 2

This case is a 13-year-old male who presented to the clinic with a history of fever for the previous 4 days. He reported fatigue, cough, vomiting and passing some red urine. He was taken to a government clinic in Thailand on the first day of fever. He was diagnosed with *P. vivax* malaria and given chloroquine plus primaquine 30mg daily for 14 days (15 mg tablets) as per the Thailand national guidelines. At home, the patient took primaquine 30mg daily for 2 days, then 30mg twice daily for 1 day (0.84mg/kg on the first 2 days, then 1.7 mg/kg for the 3 ^rd^ day). The patient then felt unwell and came to the SMRU clinic.

On physical examination, he was stable but weak with slight central cyanosis. His GCS was 15/15. Weight was 35 kg, temperature 38.8°C, HR 97 beats per minute, RR 23 respirations per minute, blood pressure 110/70 mmHg and SaO2 88% on 2LO2 by nasal cannula. On physical examination his conjunctiva and sclera were normal, heart sounds were normal, lungs clear to auscultation bilaterally, abdomen soft and without hepatosplenomegaly, and his palms were not pale or cyanotic.

At admission the malaria smear was negative, field haematocrit was 34%, and G6PD FST was deficient. Because of the cyanosis, a transcutaneous methaemoglobin measurement (Masimo®) was performed and found to be 17.1% (normal range 0–2%). He was started on Vitamin C 200mg three times daily for symptomatic methaemoglobinaemia. The primaquine was stopped and no other treatments were given.

**Figure 1.**
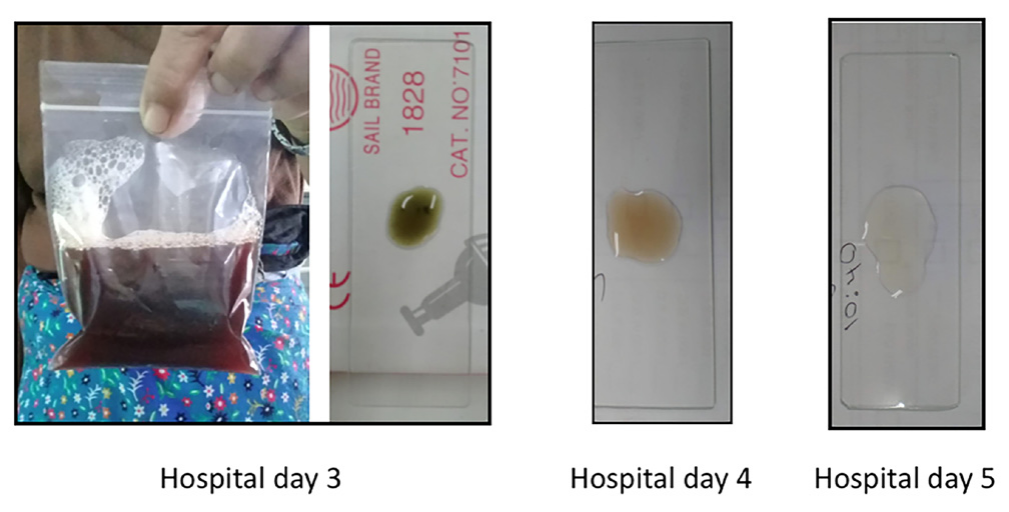
The resolution of haemoglobinuria in case 2.

On the following day (hospital day 2), results from the blood work were available ( Table 1). The CBC showed a high RBC count and low MCV. The reticulocyte count was 0.5%, which was unexpectedly low for the 4 ^th^ day of a haemolytic episode. The G6PD spectrophotometric assay confirmed the deficiency with an enzymatic activity of 0.39IU/gHb (normal value for males 8IU/gHb) and DNA analysis showed that the patient was hemizygote for G6PD Mahidol variant. Antibiotics were not prescribed as the CBC and CRP results were not suggestive of a bacterial infection. The haematocrit decreased further to 27% on hospital day 3 and was accompanied by red urine ( Figure 1). The urine sediment was negative for RBCs (consistent with intravascular haemolysis). The next day, a blood transfusion was given. Vitamin C was stopped as it is a potential exacerbator of haemolysis ^13, 14^.

Post-transfusion, his field haematocrit increased to 30% and the transcutaneous methaemoglobin values had decreased to 2.1% which were within the normal range. There were no clinical signs of acute kidney injury although his creatinine increased from 0.58 to 0.97mg/dL. The patient did not recover as quickly as expected. On hospital days 5–7, his oxygen requirement decreased to room air. He continued to have a low-grade fever (37.5°C) and this may have been due to the haemolysis. His haematocrit declined to 21%. By hospital day 8, the fever resolved spontaneously and haematocrit increased to 24%. The patient’s clinical condition was stable, so he was discharged that day by request of the family. They were counselled with the same information as for Case 1 and the G6PD deficiency card was given. For the same reasons as Case 1, the 8-week primaquine regimen was not prescribed.

## Discussion

Presented here are two G6PD deficient patients who received primaquine without G6PD testing as per the national policy of the country where the initial consultation occurred. The patient from Myanmar (Case 1) received the 8-week primaquine regimen, which should have been safe, but the doses were taken incorrectly, and severe haemolysis occurred. The patient from Thailand (Case 2) received the 14-day primaquine regimen which would have caused haemolysis even if the dose was taken correctly. In both cases haemolysis was exacerbated, by concomitant illness and a haemolytic medication ^3, 15^ and both cases had the potential to be fatal ^16^.

During treatment with primaquine (a rapidly eliminated oxidative drug) in G6PD deficient patients whose enzymatic activity is not compromised yet in reticulocytes, the haematocrit is expected to stabilize or rise the following day after the drug has been stopped given an adequate reticulocyte response ^17^. However, in both of these cases the haematocrit did not rise even after stopping primaquine. In the first case, a concomitant bacterial infection may have caused persistent haemolysis, though primaquine induced haemolysis may also be accompanied by fever and increased WBC ^18^. The second case, presented with an elevated methaemoglobin ^19^, a nearly normal haematocrit and normal reticulocyte count indicating that the bulk of haemolysis occurred after primaquine had been stopped. Administration of Vitamin C, which has been shown to cause haemolysis in G6PD deficient subjects ^13, 14, 20^ might have contributed to continued haemolysis. This patient also demonstrated reduced reticulocyte production, which may have been caused by iron deficiency or a concomitant haemoglobinopathy ^21^. Haemoglobin typing and assessment of iron deficiency could not be carried out.

The G6PD FST can give a normal result in a G6PD deficient patient who has already undergone some degree of haemolysis. Thus, a normal qualitative test result does not rule out G6PD deficiency during or shortly after a haemolytic crisis because older red blood cells with low G6PD enzymatic activity have haemolysed and younger cells with higher G6PD enzymatic activity have reached blood circulation ^22^. In this specific situation, genotyping is used to make a diagnosis of G6PD deficiency; in populations with well described variants, a well-equipped laboratory could support this approach. If genotyping is not available, the G6PD phenotypic test can be repeated later, after recovery. Transiently elevated G6PD activity can lead to an incorrect diagnosis of G6PD normality and potentially incorrect medical management.

During haemolysis, acute kidney injury from severe haemolysis and massive haematuria is a concern. Thus, using G6PD normal donor blood for transfusion can avoid further haemolysis and kidney injury. If only qualitative G6PD tests are available, ideally, the donor would be a healthy G6PD normal male. For healthy female blood donors, a quantitative G6PD test is necessary to confirm if there is enough G6PD activity (>80% normal activity) so that further haemolysis is avoided.

**Figure 2.**
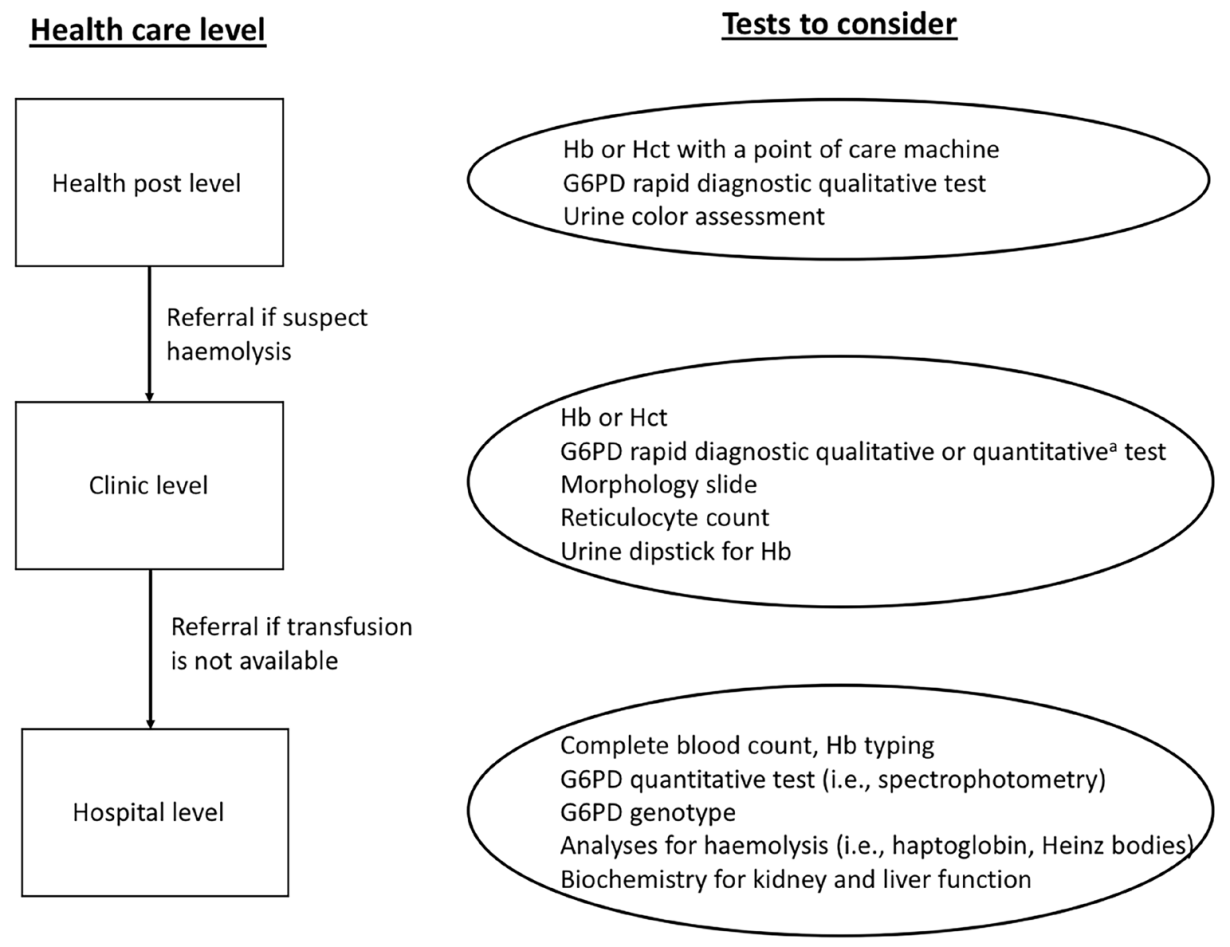
Suggested procedures when haemolysis in a G6PD deficient patient is suspected. ^a^ Currently, the STANDARD™ G6PD Test by SD Biosensor ^23, 24^ and the *Carestart*™ G6PD Biosensor by AccessBio ^25, 26^ are commercially available. The suggested procedures above apply to all G6PD genotypes. Haemoglobin (Hb), Haematocrit (Hct)

Accurate phenotypic diagnosis of G6PD deficiency and increased knowledge of what illnesses and drugs contribute to haemolysis are needed to improve the management of haemolysis. Primaquine will be used increasingly for *P. vivax* elimination, and more G6PD deficient patients will be exposed. If G6PD testing is not performed before prescribing primaquine we may see similar cases as presented here. Health care providers should consider the diagnosis of G6PD deficiency in any patient with a haemoglobin or haematocrit reduction following primaquine or any other haemolytic drug ^4^. Potential actions will depend on the health care level ( Figure 2).

To promote safe primaquine prescription for *P. vivax*, we recommend the following:

- If G6PD testing is not performed before the primaquine regimen, it is safest to supervise treatment in all treated individuals.
- If G6PD *qualitative* testing is available, counselling should be given to G6PD deficient individuals and all females about the signs and symptoms of haemolysis and anaemia, so they know when to seek care for complications. Females with intermediate G6PD activity are also at risk for haemolysis. It can be useful to supervise treatment to prevent accidental overdose and monitor for haemolysis.
- When G6PD *quantitative* testing is available, focused counselling can be given to G6PD deficient individuals and only females with intermediate G6PD activity. As above, supervised treatment further improves safety.
- In some areas, such as rural Myanmar, referral from remote health posts is not possible. In this situation, acceptable solutions are to prescribe the weekly primaquine regimen or perform point of care quantitative G6PD testing before prescribing a daily primaquine regimen and to supervise treatment in G6PD deficient or intermediate patients ^27^.

If a haemolytic complication is suspected, primaquine should be stopped. Patients should be referred as soon as possible to a facility where blood transfusion services are available. At higher levels of care, additional investigation can be done at the discretion of the health care provider ( Figure 2). When discharged, it is important to counsel the G6PD deficient patient on their disease, risks for haemolysis, and drugs to avoid and to provide a ‘G6PD deficiency card’ translated to local languages that the patient can bring to future heath care visits.

## Conclusion

With increased use of unsupervised primaquine for the elimination of *P. vivax* malaria, G6PD deficient patients (a substantial proportion in many malaria endemic populations) may be exposed to haemolysis and potentially life-threatening anaemia. Ideally, health care providers at all health care levels should counsel and/or supervise patients carefully on correct primaquine dosing to avoid accidental overdose and be watchful when administering primaquine. During haemolysis, a high suspicion of G6PD deficiency is needed. It is also important to look for other causes of haemolysis, such as infection and concomitant use of commonly prescribed potentially haemolytic drugs.

## Data availability

All data underlying the results are available as part of the article and no additional source data are required.

## Consent

Written informed consent for publication of the patients’ details and images was obtained from the guardians of the patients.
