# Supplementary material for: The impact of using primaquine without prior G6PD testing: a case series describing the obstacles to the medical management of haemolysis
Source: Wellcome Open Res. 2019 Apr 9;4:25. Originally published 2019 Feb 6. [Version 2] doi: 10.12688/wellcomeopenres.15100.2 (PMC6480970; doi:10.12688/wellcomeopenres.15100.2)
Supplement: Peer review history

# Peer review: Reviewer response for version 2

**DOI:** 10.21956/wellcomeopenres.16616.r35270

**Published:** 2019-04-17

**Authors:** Wuelton M. Monteiro

**Affiliations:** 1 Escola Superior de Ciências da Saúde, Universidade do Estado do Amazonas, Manaus, Brazil

I have read this version and believe that it can be accepted as it is.

I have read this submission. I believe that I have an appropriate level of expertise to confirm that it is of an acceptable scientific standard.

# Peer review: Reviewer response for version 2

**DOI:** 10.21956/wellcomeopenres.16616.r35271

**Published:** 2019-04-16

**Authors:** Larry A. Walker

**Affiliations:** 1 The National Center for Natural Products Research, School of Pharmacy, University of Mississippi, Mississippi, MS, USA

I appreciate the authors' responses, and I believe the revisions clarify and add to the paper's impact.

I have read this submission. I believe that I have an appropriate level of expertise to confirm that it is of an acceptable scientific standard.

# Peer review: Reviewer response for version 1

**DOI:** 10.21956/wellcomeopenres.16474.r34852

**Published:** 2019-03-07

**Authors:** Wuelton M. Monteiro

**Affiliations:** 1 Escola Superior de Ciências da Saúde, Universidade do Estado do Amazonas, Manaus, Brazil

In this manuscript, the authors describe primaquine-induced haemolysis in two G6PD Mahidol hemizygous males. Some considerations:

1. The description of cases of primaquine-induced hemolysis is not properly an original subject. What is lacking in the literature today is the knowledge of estimates of clinically relevant hemolysis among primaquine users, obtained through pharmacovigilance studies. Thus, in the introduction and discussion of the manuscript, I miss references and comparison with other series of cases of primaquine-induced hemolysis, including estimates of the primaquine-induced hemolysis rate among drug users. See: Recht *et al.* (2014 ^1^), Monteiro *et al.* (2014 ^2^) and Brito-Sousa *et al.* (2019 ^3^).
2. Genetic variants associated to PQ-induced hemolysis are a matter of debate. Thus, include a discussion of those variants, whose presence may result in dangerous or even potentially life-threatening hemolysis, including now Mahidol. See: Recht *et al.* (2014 ^1^) and Monteiro *et al.* (2016 ^4^).
3. The 8-week course of PQ under supervision was indicated as the possible alternative radical cure treatment for these cases, but no further information on the outcome was presented. Was it employed? Was it safety checked?

I have read this submission. I believe that I have an appropriate level of expertise to confirm that it is of an acceptable scientific standard, however I have significant reservations, as outlined above.

## References

[1] RechtJAshleyEWhiteN: Safety of 8-aminoquinoline antimalarial medicines. *World Health Organization*.2014; Reference source

[2] MonteiroWMFrancaGPMeloGCQueirozALBritoMPeixotoHMOliveiraMRRomeroGABassatQLacerdaMV: Clinical complications of G6PD deficiency in Latin American and Caribbean populations: systematic review and implications for malaria elimination programmes. *Malar J*.2014;13: 10.1186/1475-2875-13-70 70 10.1186/1475-2875-13-70 24568147PMC3938641

[3] Brito-SousaJDSantosTCAvalosSFontechaGMeloGCValFSiqueiraAMAlecrimGCBassatQLacerdaMVGMonteiroWM: Clinical Spectrum of Primaquine-induced Hemolysis in G6PD Deficiency: A Nine-Year Hospitalization-Based Study from the Brazilian Amazon. *Clin Infect Dis*.2019; 10.1093/cid/ciz122 10.1093/cid/ciz122 30753364

[4] MonteiroWMoura-NetoJRechtJBassatQLacerdaM: Fatal Primaquine-Induced Hemolysis in a Patient WithPlasmodium vivax Malaria and G6PD A(−) Variant in the Brazilian Amazon. *Clinical Infectious Diseases*.2016;62(9) : 10.1093/cid/ciw039 1188.1-1188 10.1093/cid/ciw039 26826373

# Response

**Published:** 2019-04-02

**Authors:** Cindy Chu

**Affiliations:** Faculty of Tropical Medicine, Shoklo Malaria Research Unit, Mahidol University, Thailand

We would like to thank Reviewer 2 for suggesting further references and providing comments that initiate further contemplation on this manuscript in the public domain.

1. *The description of cases of primaquine-induced hemolysis is not properly an original subject. What is lacking in the literature today is the knowledge of estimates of clinically relevant hemolysis among primaquine users, obtained through pharmacovigilance studies. Thus, in the introduction and discussion of the manuscript, I miss references and comparison with other series of cases of primaquine-induced hemolysis, including estimates of the primaquine-induced hemolysis rate among drug users. See: Recht et al.(2014 ^1^), Monteiro et al.(2014 ^2^) and Brito-Sousa et al.(2019 ^3^).*

******RESPONSE:** This case series focuses on the haemolysis caused by patients taking unsupervised primaquine incorrectly. But as Reviewer 2 points out, G6PD deficient patients taking daily primaquine (presumably as prescribed) are also at risk for hemolysis. To raise this issue in the manuscript two changes have been made. The last sentence of the second paragraph in the introduction has been amended to “This means that undiagnosed G6PD deficient patients have the potential to develop primaquine induced haemolysis, especially with *daily *regimens.” A reference suggested by the reviewer has been added. The last sentence at the end of the first paragraph of the discussion has amended to highlight the risk of mortality in these patients. “In both cases haemolysis was exacerbated, by concomitant illness …. *and both cases also had the potential to be fatal*.” A reference suggested by the reviewer has been added.

2. *Genetic variants associated to PQ-induced hemolysis are a matter of debate. Thus, include a discussion of those variants, whose presence may result in dangerous or even potentially life-threatening hemolysis, including now Mahidol. See: Recht et al. (2014 ^1^) and Monteiro et al. (2016 ^4^).*

******RESPONSE:** ****This comment raises a few thoughts. Firstly, there is very little known about the associated PQ-induced haemolytic risk in the vast majority of G6PD variants. Primaquine challenge clinical trials in the past were mainly carried out in hemizygous A- subjects (Tarlov *et al.*, 1962) and rarely in subjects from Sardinia, presumably with Mediterranean variant (Pannacciulli, 1965). In the last few years, clinical trials have been carried out in subjects with Mahidol variant, but data come from treatment in heterozygous women with normal phenotype by FST. Secondly, some variants such as Mahidol and African A-, are described as ‘mild’ based on the WHO classification. The data informing the WHO classification are based on small numbers of analyzed subjects (sometimes just one per variant) often poorly characterized (genotyping in pre-PCR era, non-standardized biochemical and enzymatic analysis), and cannot be used to indicate the associated hemolytic risk. This means that the evidence available to date on G6PD variants, described as ‘mild’ by the WHO classification, in fact show that hemolysis does occur and is life threatening. As much as we would like to include a discussion on this important complicated topic in the manuscript, it is not the main point of the manuscript. The recommendations in the discussion apply to all genotypes and no differentiation is made. However, to improve clarity, we have added a footnote to Figure 2 that states *“The suggested procedures above apply to all G6PD genotypes*”.

3. *The 8-week course of PQ under supervision was indicated as the possible alternative radical cure treatment for these cases, but no further information on the outcome was presented. Was it employed? Was it safety checked?*

******RESPONSE:** ****We have added the outcome to the case presentations. In both cases weekly primaquine was not given because the close medical supervision was not possible.

***Is the background of the cases’ history and progression described in sufficient detail?*** *Partly.*

******RESPONSE:** ****We have added more detail to the cases to address the reviewer comment on case history and progression.

***Are enough details provided of any physical examination and diagnostic tests, treatment given and outcomes?*** *Partly.*

******RESPONSE:** ****We have added to the manuscript an explanation of the patient outcomes.

# Peer review: Reviewer response for version 1

**DOI:** 10.21956/wellcomeopenres.16474.r34850

**Published:** 2019-03-01

**Authors:** Larry A. Walker

**Affiliations:** 1 The National Center for Natural Products Research, School of Pharmacy, University of Mississippi, Mississippi, MS, USA

The authors present two cases which further illustrate the dangers of using PQ in populations where G6PD status is unknown, especially in unsupervised treatment settings. These two reports convey details that emphasize the complexity of the problems of hemolysis etiology, treatment and recovery, as encountered by practitioners in malaria endemic countries. In particular, both patients took PQ incorrectly due to poor understanding of instructions. But the laboratory findings and recovery course were complicated by other medications, concurrent illness, and timing of presentation to the clinic. The authors have done an excellent job of framing the history and clinical details, and then interpreting these in their relevance to current knowledge and implications for future utilization of PQ. They rightly point out the urgent need for better training of health workers in recognizing possible PQ-induced hemolysis, and in specific instruction of patients regarding the importance of strict adherence to the drug regimen; this should entail careful attention to literacy and language barriers, which seemed to be limiting in these cases.

 I have the following comments regarding the article:

1. The authors have pointed out the constraints of relying on G6PD activity tests for diagnosing G6PD deficiency after a hemolytic episode has already ensued. But it seems worthwhile to clarify this in the recommendations (in the discussion on Figure 2), which might be construed as employing the qualitative tests “in any patient with a haemoglobin or haematocrit reduction following primaquine or any other haemolytic drug” in the prior sentence. This might be misunderstood by casual readers. 
2. Regarding the time course of the recovery in hemoglobin, mentioned in paragraph two of the Discussion, some further development on this issue would be helpful. The authors state, “During treatment with primaquine (a rapidly eliminated oxidative drug) in G6PD abnormal patients, the haematocrit is expected to stabilize or rise the following day after the drug has been stopped. ^14^” This reference is the WHO Guidelines, but I wonder if this quick recovery has a solid basis in clinical evidence. While evidence has suggested the “self-limiting” character of the hemoglobin drop, and even recovery with continued dosing (a “resistance phase”, article reference 19), I wonder if these would be expected so rapidly. In Rueangweerayut *et al. *(2017 ^1^) the modest drop in hemoglobin with 14d PQ dosing in heterozygous Mahidol females was sustained for the 14 days dosing, though recovered by 21 days. The data from Kheng *et al.* (article reference 3), suggest that after a single 45 mg dose of PQ in Viangchan deficients, the decline in hemoglobin was seen on day 1 and sustained for a week – though it did recover during subsequent weekly doses. It seems like the recovery picture may be an interplay between the oxidative stress insult (due to continued drug dosing or to persistent metabolite(s) recycling?) and the erythropoietic recovery mechanism. I may be off base, but I think it would be of interest to have the authors’ perspectives on these.
3. Further to time course issues, the development of the hemolytic response in these two cases is perhaps worthy of note. Though the regimens prescribed were presumably different, the patients took 30 mg/day for 4 days (case 1) and 30mg/day for 2 days and 60 mg on the third day (case 2), when they developed serious symptoms. This, along with other studies, suggests that it takes 2-3 days for the hemolytic clinical picture to fully develop after dosing. In Rueangweerayut *et al.* (2017 ^1^), with 15 mg/d it took 3-4 days to elicit a hemoglobin drop. But with the higher weekly dose of 45 mg in the Cambodian study, a drop in hemoglobin was observed on the first day (article reference 3). Since neither the drop in hemoglobin, nor the recovery, are related to the parent drug, it might again suggest some persistent insult from a red cell metabolite recycling. Or perhaps there is some lag time in the development of the hemolytic picture because of other dynamics to reach the clinically severe stages? The authors’ insights here, if any, might be useful. 
4. The apparent intravascular hemolysis observed here is perhaps worthy of note. How often is this apparent in such cases? In some of the animal studies, and in a number of case reports, there appears to be erythrocyte damage and clearance without intravascular hemolysis. Is this perhaps related to severity of deficiency in G6PD activity?
5. In the first paragraph of the discussion, the last sentence appears to be incorrectly worded. I am perhaps unfamiliar with this use of “mediation”. Or perhaps it should be “and another haemolytic medication”? Also, do these references (article references 3 and 13) support the last sentence, or the one prior?

I have read this submission. I believe that I have an appropriate level of expertise to confirm that it is of an acceptable scientific standard, however I have significant reservations, as outlined above.

## References

[1] RueangweerayutRBanconeGHarrellEJBeelenAPKongpatanakulSMöhrleJJRousellVMohamedKQureshiANarayanSYubonNMillerANostenFHLuzzattoLDuparcSKleimJPGreenJA: Hemolytic Potential of Tafenoquine in Female Volunteers Heterozygous for Glucose-6-Phosphate Dehydrogenase (G6PD) Deficiency (G6PD Mahidol Variant) versus G6PD-Normal Volunteers. *Am J Trop Med Hyg*.2017;97(3) : 10.4269/ajtmh.16-0779 702-711 10.4269/ajtmh.16-0779 28749773PMC5590573

# Response

**Published:** 2019-04-02

**Authors:** Cindy Chu

**Affiliations:** Faculty of Tropical Medicine, Shoklo Malaria Research Unit, Mahidol University, Thailand

We would like to thank Reviewer 1 for comments and dialogue on this manuscript in the public domain. 

1. *The authors have pointed out the constraints of relying on G6PD activity tests for diagnosing G6PD deficiency after a hemolytic episode has already ensued. But it seems worthwhile to clarify this in the recommendations (in the discussion on Figure 2), which might be construed as employing the qualitative tests “in any patient with a haemoglobin or haematocrit reduction following primaquine or any other haemolytic drug” in the prior sentence. This might be misunderstood by casual readers.*

******RESPONSE**: The transition of this paragraph was amended to separate the idea of constraints of relying on G6PD tests after a hemolytic episode and how to improve the safety of prescribing primaquine. A new paragraph was inserted and the opening sentence of the new paragraph was clarified to read “To promote safe primaquine prescription for *P. vivax*, …..” 

2. *Regarding the time course of the recovery in hemoglobin, mentioned in paragraph two of the Discussion, some further development on this issue would be helpful. The authors state, “During treatment with primaquine (a rapidly eliminated oxidative drug) in G6PD abnormal patients, the haematocrit is expected to stabilize or rise the following day after the drug has been stopped. ^14^” This reference is the WHO Guidelines, but I wonder if this quick recovery has a solid basis in clinical evidence. While evidence has suggested the “self-limiting” character of the hemoglobin drop, and even recovery with continued dosing (a “resistance phase”, article reference 19), I wonder if these would be expected so rapidly. In Rueangweerayut et al. (2017) the modest drop in hemoglobin with 14d PQ dosing in heterozygous Mahidol females was sustained for the 14 days dosing, though recovered by 21 days. The data from Kheng et al. (article reference 3), suggest that after a single 45 mg dose of PQ in Viangchan deficients, the decline in hemoglobin was seen on day 1 and sustained for a week – though it did recover during subsequent weekly doses. It seems like the recovery picture may be an interplay between the oxidative stress insult (due to continued drug dosing or to persistent metabolite(s) recycling?) and the erythropoietic recovery mechanism. I may be off base, but I think it would be of interest to have the authors’ perspectives on these.*

******RESPONSE:** This comment raises 2 important questions. The first is about the attenuation of the haemolysis in G6PD deficient individuals despite continuing primaquine and the second is about hematologic recovery. We can share our thoughts and clinical experiences: 

Question 1: There is little literature on acute hemolysis during weekly primaquine treatment in G6PD deficient cohorts and even less in those who inadvertently were administered daily doses. Historical data in certain G6PD deficient individuals show that when primaquine doses are continued, the hemolysis becomes self-limited and haematocrit will rise, presumably due to increased reticulocyte production and a younger RBC population (G6PD activity is higher in reticulocytes). Additionally, in Rueangweerayut *et al. *(2017) and Chu *et al*. (2016), G6PD heterozygous females who were diagnosed as G6PD normal on a qualitative test demonstrated that haemoglobin recovery begins around day 7 despite continued primaquine dosing. Though, it does take more time to reach full recovery. 

Question 2: In our clinical experience with *P. vivax *patients, when hemolysis with severe anemia occurs and primaquine is stopped immediately (usually between days 3-6 after the first dose), we see the hematocrit rise in the following few days (along with a rise in reticulocytes). We do not have the experience of stopping primaquine at days 0-2 after the first dose when the reticulocyte response may not yet have increased sufficiently enough for a large effect on the RBC population. Our thoughts are that the recovery picture is related to the physiologic (erythropoietic) response of the individual and concomitant diseases that individual may have (i.e. concurrent illness, hemoglobinopathies). Moreover, the G6PD activity in reticulocytes has been shown to be normal in Mahidol reticulocytes (Bancone, 2017) and is presumably so in the A- variant, while it is expected to be already compromised in mutations such as Mediterranean (Piomelli, 1958). This would affect haematologic recovery even if the reticulocyte response is adequate. Further data on primaquine metabolism may add to these assumptions. To clarify this issue in the manuscript, we have amended a sentence in the second paragraph of the discussion to read “During treatment with primaquine (a rapidly eliminated oxidative drug) in G6PD abnormal patients whose enzymatic activity is not compromised yet in reticulocytes, the hematocrit is expected or stabilize or rise the following day after the drug has been stopped *given an* *adequate reticulocyte response*.” We do not have other references aside from our unpublished clinical experience and thus will continue using the WHO reference.

3. *Further to time course issues, the development of the hemolytic response in these two cases is perhaps worthy of note. Though the regimens prescribed were presumably different, the patients took 30 mg/day for 4 days (case 1) and 30mg/day for 2 days and 60 mg on the third day (case 2), when they developed serious symptoms. This, along with other studies, suggests that it takes 2-3 days for the hemolytic clinical picture to fully develop after dosing. In Rueangweerayut et al. (2017 ^1^), with 15 mg/d it took 3-4 days to elicit a hemoglobin drop. But with the higher weekly dose of 45 mg in the Cambodian study, a drop in hemoglobin was observed on the first day (article reference 3). Since neither the drop in hemoglobin, nor the recovery, are related to the parent drug, it might again suggest some persistent insult from a red cell metabolite recycling. Or perhaps there is some lag time in the development of the hemolytic picture because of other dynamics to reach the clinically severe stages? The authors’ insights here, if any, might be useful.*

******RESPONSE:** This question is in regard to the development of hemolysis while on an 8-aminoquinoline. In Rueangweerayut *et al. *(2017) single dose tafenoquine was used in some G6PD intermediate healthy subjects whereas in Kheng et al. (2015) primaquine weekly dose was used in G6PD deficient patients with *P. vivax *infection. Peak concentrations of primaquine are reached ~3 hours after dosing whereas peak concentrations of tafenoquine are reached at ~20 hours. The time to development of the full hemolytic picture in these studies may be affected by *P. vivax *infection, G6PD activity (greater proportion of older and deficient cells in deficient subjects so hemolysis occurs earlier), and drug concentrations. Of course, we do not have enough knowledge of primaquine or tafenoquine metabolism to know if there are active metabolites that are recycled and cause prolonged oxidative effects.

4. *The apparent intravascular hemolysis observed here is perhaps worthy of note. How often is this apparent in such cases? In some of the animal studies, and in a number of case reports, there appears to be erythrocyte damage and clearance without intravascular hemolysis. Is this perhaps related to severity of deficiency in G6PD activity?*

******RESPONSE:** ****It is very difficult to answer this question; cases of evident haemoglobinuria are usually reported while increased levels of bilirubin or free hemoglobin in blood can be detected almost only by specific laboratory investigations. As suggested in Luzzatto & Seneca (2014), 8-aminoquinoline-induced hemolysis is both intravascular and extravascular, with the former happening in more severely affected RBCs and the latter in less damaged RBCs. We do not know whether this might be related to the severity of enzymatic deficiency.

5. *In the first paragraph of the discussion, the last sentence appears to be incorrectly worded. I am perhaps unfamiliar with this use of “mediation”. Or perhaps it should be “and another haemolytic medication”? Also, do these references (article references 3 and 13) support the last sentence, or the one prior?*

******RESPONSE:** The word ‘mediation’ has been corrected to ‘medication’. In reference 3, a G6PD deficient male who received 45 mg primaquine developed severe anaemia requiring a blood transfusion. He had also received Ciprofloxacin (a hemolytic agent), which would support the idea that haemolysis could be exacerbated by concomitant drugs in addition to an 8-aminoquinoline. There are case reports of large doses of Vitamin C causing haemolysis but not in a normal dose concomitantly with another haemolytic agent. We have added a reference (Monteiro *et al.*, 2016) that further supports these sentences.
